# Experimental Methodologies and Evaluations of Computer-Aided Drug Design Methodologies Applied to a Series of 2-Aminothiophene Derivatives with Antifungal Activities

**DOI:** 10.3390/molecules17032298

**Published:** 2012-02-24

**Authors:** Luciana Scotti, Marcus Tullius Scotti, Edeltrudes de Oliveira Lima, Marcelo Sobral da Silva, Maria do Carmo Alves de Lima, Ivan da Rocha Pitta, Ricardo Olímpio de Moura, Jaismary Gonzaga Batista de Oliveira, Rayssa Marques Duarte da Cruz, Francisco Jaime Bezerra Mendonça

**Affiliations:** 1 Centro de Biotecnologia, Universidade Federal da Paraíba, João Pessoa 50670-910, PB, Brazil; Email: marcelosobral@ltf.ufpb.br (M.S.S.); 2 Departamento de Engenharia e Meio Ambiente, Universidade Federal da Paraíba, Campus IV, Rio Tinto 58297-000, PB, Brazil; Email: mtscotti@gmail.com; 3 Laboratório Micologia, Departamento de Ciências Farmacêuticas, Centro de Ciências da Saúde, Universidade Federal da Paraíba, João Pessoa 50670-910, PB, Brazil; Email: edelolima@yahoo.com.br; 4 Laboratório de Planejamento e Síntese de Fármacos, Departamento de Antibióticos, Universidade Federal de Pernambuco, Recife 50670-910, PE, Brazil; Email: nenalima.mariadocarmo@gmail.com (M.C.A.L.); irpitta@gmail.com(I.R.P.); 5 Laboratório de Síntese e Vetorização de Moléculas, Departamento de Ciências Biológicas, Universidade Estadual da Paraíba, Rua Horácio Trajano de Oliveira s/n, Cristo Redentor, João Pessoa 58070-450, PB, Brazil; Email: ricardo.olimpiodemoura@gmail.com (R.O.M.); jaismaryy@gmail.com (J.G.B.O.); rayssaduarte@gmail.com (R.M.D.C.)

**Keywords:** 2-aminothiophene derivatives, antifungal activity, molecular modelling, computer-aided drug design, density functional theory

## Abstract

Fifty 2-[(arylidene)amino]-4,5-cycloalkyl[*b*]thiophene-3-carbonitrile derivatives were screened for their *in vitro *antifungal activities against *Candida krusei* and *Cryptococcus neoformans*. Based on experimentally determined minimum inhibitory concentration (MIC) values, we conducted computer-aided drug design studies [molecular modelling, chemometric tools (CPCA, PCA, PLS) and QSAR-3D] that enable the prediction of three-dimensional structural characteristics that influence the antifungal activities of these derivatives. These predictions provide direction with regard to the syntheses of new derivatives with improved biological activities, which can be used as therapeutic alternatives for the treatment of fungal infections.

## 1. Introduction

Systemic fungal infections have gradually increased over the last three decades, leading to considerable rates of morbidity and mortality, principally due to the low effectiveness of available medications and the development of resistant strains. Immunocompromised patients, such as those with previous diseases like tuberculosis, cancer, diabetes and AIDS and patients undergoing chemotherapy or who have had transplants, are more susceptible to opportunistic fungal infections, which often prove fatal [1].

Conventional treatment regimes for diseases caused by fungi are based on only a few chemotherapies (*i.e.*, azoles and polyenes), and these are not completely effective and present serious problems related to their narrow spectrum, high toxicity, low potency (they are generally fungistatic) and inadequate pharmacokinetic properties. Taken together, these characteristics result in many cases of microbial resistance, making treatment even more difficult [2].

Thus, there is a clear need to develop new antifungal agents as therapeutic alternatives for the control of fungal infections. In this context, compounds derived from heterocyclic thiophenes have emerged as promising antifungal drugs [3,4,5,6,7,8,9,10], some of which have become commercially available (for example, sertaconazol, a benzothiophene derivative).

The microorganisms *Candida krusei* and *Criptococcus neoformans* were selected for the present study because they presented better profiles of antifungal sensitivity compared to other 2-amino-thiophene derivatives previously synthesised by our group [10]. Furthermore, these microorganisms have additional medical relevance in that *Candida krusei* shows natural resistance to the standard medication fluconazole, one of the most widely used antifungals in clinical medicine. The same microorganism shows reduced sensitivity to both flucytosine and amphotericin B [11,12,13] and causes greater mortality than the species *C. albicans* [14] and *C. neoformans* due to its unusual ability to evolve into meningitis. *Candida krusei* and *Criptococcus neoformans* are also important markers for HIV infection [15,16].

**Figure 1:**
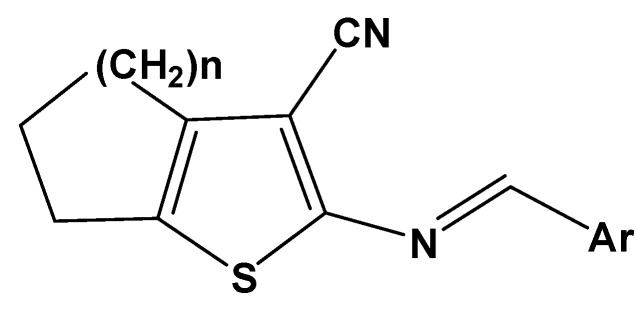
General structure of the studied 2-aminothiophenes.

This study investigates a series of fifty 2-[(arylidene)-amino]-4,5-cycloalkyl[*b*]thiophene-3-carbonitrile derivatives (generically called 2-aminothiophenes, Figure 1). After experimentally assessing their antifungal activities *in vitro *by testing their sensitivities to the fungal species *Candida krusei* and *Cryptococcus neoformans,* the derivatives were subjected to molecular modelling, chemometric tools and QSAR-3D in order to extract structural information relating to their antifungal properties [17,18,19,20,21]. 

## 2. Results and Discussion

### 2.1. Synthesis and Antifungal Activity

The fifty 2-aminothiophenes evaluated in this study (Figure 1) were previously synthesised and spectroscopically characterised by this group [10,22,23] using the classic Gewald reaction [24] followed by substitution (via condensation) with different aromatic aldehydes. Table 1 presents the chemical structures of the 50 derivatives studied (compounds **1**–**50**) as well as their minimum inhibitory concentrations (MIC) expressed in μg/mL (referring to their antifungal activities with respect to the fungi *Candida krusei* and *Cryptococcus neoformans*).

**Table 1:** Anti-*Candida* and anti-*Criptococcus* activities and chemical formulas of the studied 2-aminothiophene derivatives.

Comp.	n	Ar	MIC (μg/mL)	
			*C. neoformans *ICB59	*Candida krusei *LM13
**1**	1	Ph	2,500	1,250
**2**	1	4-NO_2_-Ph	1,250	2,500
**3**	1	2-Thiophenyl	156	>10,000
**4**	1	2-Indole	1,250	>10,000
**5**	1	4-F-Ph	2,500	>10,000
**6**	1	4-Br-Ph	2,500	>10,000
**7**	1	4-Morpholine	312	>10,000
**8**	1	4-OEt-Ph	2,500	>10,000
**9**	1	4-Cl-Ph	5,000	2,500
**10**	1	4-OMe-Ph	1,250	2,500
**11**	1	2,3-Cl-Ph	156	>10,000
**12**	1	3,4-Cl-Ph	>10,000	5,000
**13**	1	2,4-Cl-Ph	78	78
**14**	1	3,4,5-OMe-Ph	156	78
**15**	1	2-OMe,5-Br-Ph	156	78
**16**	1	8-Quinoline	156	5,000
**17**	2	4-NO_2_-Ph	1,250	128
**18**	2	2-Indole	78	>10,000
**19**	2	4-OBz-Ph	>10,000	625
**20**	2	4-Br-Ph	>10,000	>10,000
**21**	2	4-Cl-Ph	>10,000	>10,000
**22**	2	4-OMe-Ph	156	1,250
**23**	2	4-F-Ph	5,000	625
**24**	2	2,3-Cl-Ph	156	156
**25**	2	3,4-Cl-Ph	1,250	312
**26**	2	2,4-Cl-Ph	2,500	>10,000
**27**	2	3,4,5-OMe-Ph	156	2,500
**28**	2	2-OMe,5-Br-Ph	10,000	10,000
**29**	2	4-Pyrrolidine-Ph	2,500	2,500
**30**	2	3-Thiophenyl	2,500	>10,000
**31**	2	3,4-OBz-Ph	2,500	1,250
**32**	2	8-Quinoline	78	625
**33**	2	4-Morpholine-Ph	2,500	2,500
**34**	3	4-Me-Ph	156	2,500
**35**	3	4-iPr-Ph	1,250	2,500
**36**	3	Ph	>10,000	2,500
**37**	3	4-NO_2_-Ph	10,000	625
**38**	3	2-indole	312	2,500
**39**	3	4-OBz-Ph	10,000	>10,000
**40**	3	4-Br-Ph	2,500	5,000
**41**	3	4-Cl-Ph	1,250	2,500
**42**	3	4-OMe-Ph	312	2,500
**43**	3	4-OEt-Ph	5,000	2,500
**44**	3	3,4-Cl-Ph	1,250	2,500
**45**	3	2,4-Cl-Ph	>10,000	2,500
**46**	3	3,4,5-OMe-Ph	1,250	1,250
**47**	3	2-OMe,5-Br-Ph	10,000	5,000
**48**	3	3-Thiophenyl	2,500	>10,000
**49**	3	8-Quinoline	78	625
**50**	3	4-Et-Ph	2,500	2,500

### 2.2. Electronic Surfaces

**Figure 2:**
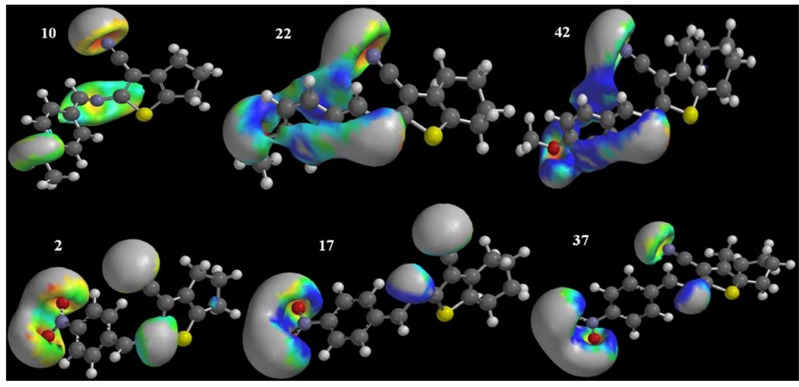
Ionisation potentials obtained for compounds **10**, **22**, **42**, **2**, **17** and **37**.

The density functional theory of electrostatic potential and ionisation potential and the HOMO and LUMO orbital surface were determined for each compound. Figure 2 shows some striking examples regarding ionisation potential. To obtain and compare surfaces’ ionisation potentials, we adopted a single band (red 14800–215000 blue). To evaluate the electronic surfaces, the molecules were represented by the tube model. Specific atoms are represented by colours as follows: carbon (grey), oxygen (red) and hydrogen (white). Using the Spartan program, these electronic-surface studies revealed that derivatives with the same aryl substituent linked to the 2-amino portion show significant differences in their reactivities (compounds **10**, **22** and **42**–4-methoxyphenyl substituent; **2**, **17** and **37**–4-nitrophenyl substituent), depending on the type of cycloalkyl ring attached to each thiophene nucleus. The cyclopenta[*b*]thiophene derivatives exhibited lower reactivities compared to the cyclohexa- and cyclohepta[*b*]thiophene derivatives. This difference in reactivities was demonstrated principally by comparing the ionisation surface potentials, with cyclopenta[*b*]thiophenes exhibiting negative ionisation potentials (red) and cyclohexa- and cyclohepta-[*b*]thiophenes exhibiting positive ionisation potentials (blue) (Figure 2).

**Figure 3:**
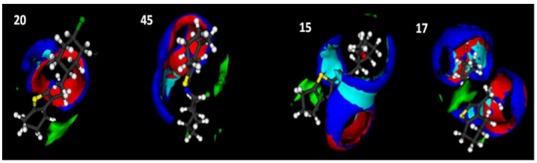
Maps of interaction with the probes H2O, N1 and O obtained for inactive compounds **20** and **45** and for active compounds **15** and **17**.

Using the VolSurf+ program, the compounds were subjected to the GRID force field, and their interactions with the following probes were evaluated: H_2_O (light blue), O (red), N (dark blue) and DRY (green) (Figure 3).

The interaction maps generated with the GRID force field show that, independent of the reactivities and electronic behaviours exhibited by cyclopenta-, cyclohexa- and cyclohepta-[*b*]thiophenes, the inactive compounds (for example: compounds **20** and **45**) had little or no interaction with the H_2_O probe. In this study, the cyclopenta[*b*]thiophene derivatives exhibited greater interactions with the H_2_O probe and better antifungal profiles, indicating that increases in water solubility correlate with increases in antifungal activity for these derivatives. 

At first glance, the cyclopenta[*b*]thiophenes appear more active, leading us to believe that they are also more water soluble. This belief was confirmed by subsequent analyses. Thus, we conclude that more active compounds exhibit greater interactions with H_2_O.

### 2.3. Chemometric Studies Using the Program VolSurf+

After conformational analyses, data obtained for the fifty 2-aminothiophene derivatives shown in Table 1 were subjected to chemometric analyses [17,18,19] (PCA and CPCA) using the VolSurf+ program for Windows [25,26].

#### 2.3.1. CPCA (Consensus PCA)

A preliminary exploratory analysis, CPCA, which considered 128 independent variables or descriptors, was developed. Pre-processing (autoscaling) of data was performed, and 13 blocks of descriptors were calculated. Table 2 and Table 3 present the values obtained for the variance in anti-*Candida *and anti-*Cryptococcus *activities.

**Table 2:** Results of CPCA analyses of anti-*Candida *activities.

PC	% explained variance from original data
1	41.26
2	22.69
3	3.06
4	1.05
5	0.00

**Table 3:** Results of CPCA analyses of anti-*Cryptococcus *activities.

PC	% explained variance from original data
1	45.03
2	20.11
3	4.00
4	0.98
5	0.00

**Figure 4:**
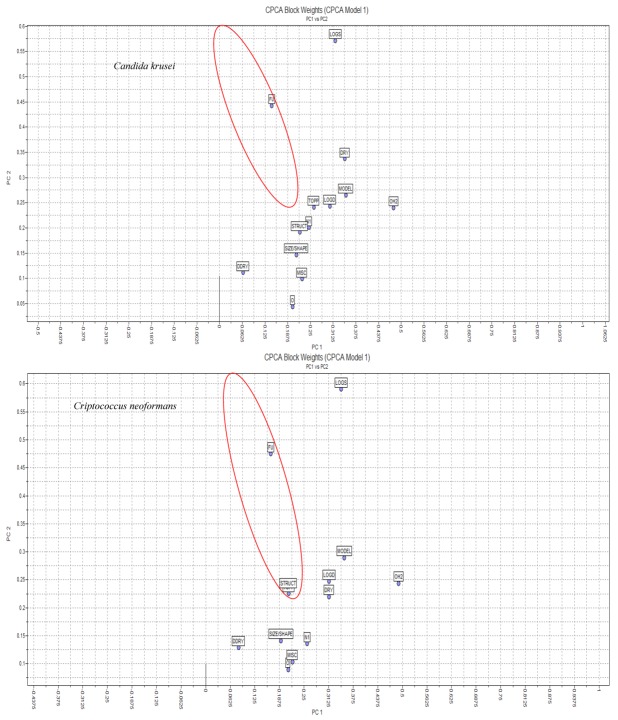
The relationship between the weights of the blocks of descriptors and the PC1 and PC2 components of the CPCA calculated using activities against *C. krusei* and *C. neoformans*.

The blocks of descriptors that presented higher weights are shown in the Figure 4 graphs, which were constructed taking into account the orthogonal properties of the CPCA: LogS (ADME descriptors) algorithm and H_2_O, resulting in a total of 34 variables.

Regarding CPCA formalism, 128 independent variables were considered, and no biological data were used as input to the model. The orthogonal properties of the CPCA algorithm were explored. The use of CPCA in decentralised process monitoring and diagnosis is derived from regular PCA scores and residuals. Two significant principal components (PCs) were found using a cross-validation technique, explaining more than 60% of the total variance in anti-*Candida* and anti-*Criptococcus* activities (Table 3 and Table 4). 

In these CPCA studies, we observed super block-weights and, comparing several blocks of descriptor variables measured on the same objects, became aware of the important influence that each block has on the associated calculations. Thirteen blocks of descriptors were calculated, and their weights were plotted considering two factors: PC1 and PC2. 

**Table 4:** Results of PCA analyses of anti-*Candida* activities.

PC	% explained variance from original data
1	43.77
2	20.58
3	2.33
4	1.71
5	0.09

As shown in Figure 4, the LogS and H_2_O blocks presented significant weights in relation to PC2 and PC1, respectively. As previously mentioned, the CPCA algorithm is essentially equivalent to the regular PCA, but new definitions of blocks and variables of larger contributions were investigated with PCA.

#### 2.3.2. PCA

With regard to the interaction of 3D structures and a GRID force field, PCA results were obtained using the H_2_O and LogS probes. Thirty-four descriptors were calculated. The data were autoscaled (pre-processed). Once again, for both activities, approximately 60% of the total variance was explained by the PC1 and PC2 components (Table 4 and Table 5).

**Table 5:** Results of PCA analyses of anti-*Cryptococcus* activities.

PC	% explained variance from original data
1	39.80
2	22.75
3	3.56
4	1.00
5	0.08

**Figure 5:**
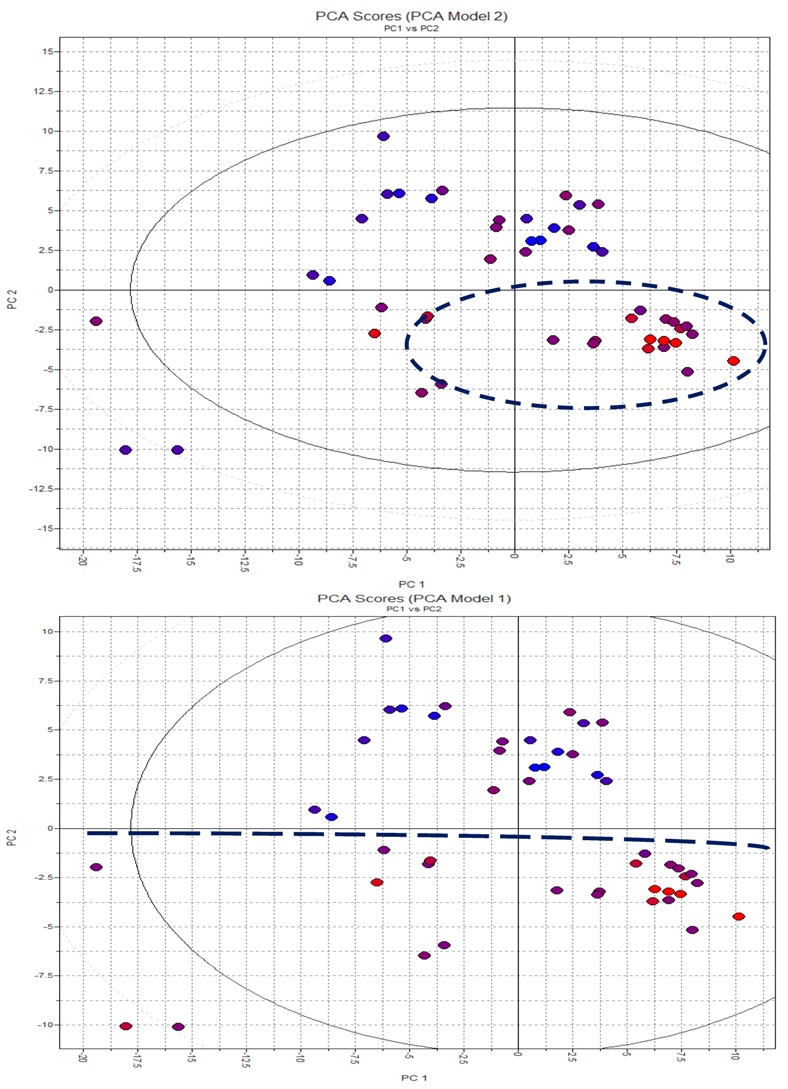
Positions of objects in relation to PC1 and PC2 components. Blue represents more active compounds, lilac represents compounds that are moderately active, and pink represents less active compounds.

Good separations were observed between active and inactive compounds for both anti-*Candida *activities and anti-*Cryptococcus *activities (Figure 5). The PCA studies considered the 3D interaction energies calculated using the LogS and H_2_O probes in a GRID force field. The PCA method also served to refine the data. Thirty-four total descriptors were calculated. Using the *leave-one-out* (LOO) cross-validation technique (Table 4 and Table 5), the PC1 and PC2 components captured approximately 60% of the total variance from the original data. Active and inactive compounds were well distinguished (see Figure 5). Defined clusters of active and inactive compounds were observed when the LogS and H_2_O VolSurf descriptors were used. 

The VolSurf descriptors give the possibility of representing the molecular interaction regions of interest graphically, a big advantage in pharmacological studies and are independent of the molecular orientation within the grid. The main advantage of the GRID method is the great variety of available probes, represented by several functional groups among them there are probes that can both accept and donate a hydrogen bond (e.g., water), probes that cannot turn around (e.g., carbonyl probe), and a hydrophobic probe. These results indicate the strong predictability of the model. 

The presence of descriptors indicating the following pharmacokinetic properties influenced antifungal activity: CACO2, SKIN, L1, 2GS, SOLY, LOGS10, LOGS11. In addition, FLEX_RB highlights conformational characteristics. These abbreviations are defined as follows:

- SOLY-intrinsic solubility,
- LOGS10 and 11-intrinsic solubilities calculated at different pHs (pH = 10 and pH = 11),
- CACO2 and SKIN-permeability/solubility in skin cells and CACO2,
- L1 and 2 GS-solubility for Legendre-polynomials that are used to differentiate solubility between similar compounds,
- FLEX_RB–A parameter related to flexibility and the number of rotational links. The Flex descriptor represents the maximum flexibility of a molecule. It is the result of the average of the differences between the maximum and minimum distance of every atom with the others searched on 50 random conformers). Flex_RB descriptor is the ratio between Flex and the number of rotable bonds.

Most of these descriptors relate to the influence of solubility on antifungal activity, consistent with the interaction maps for the GRID force field. Studies regarding the toxicity of these compounds are underway; in the future, we expect to use these active agents in formulations that have higher solubilities.

### 2.4. QSAR-3D and PLS

**Figure 6:**
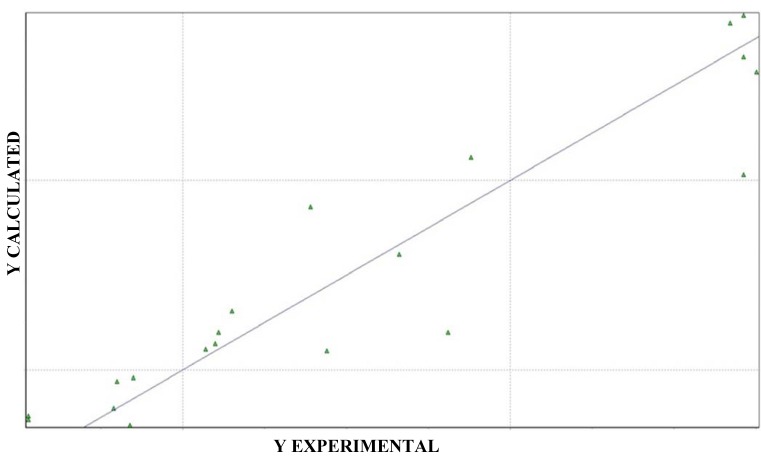
Graph of experimental *versus* calculated activity values, predicted by Equation (1).

Significant results were obtained using the antifungal activity against *C. krusei*. The best model and its statistical indices are presented in equations (1) and (2). Figure 6 reports the arrangement of selected objects to form the training set (experimental *versus* calculated activity values).

−log(MFC) = + 15.99 (± 5.34) H7m − 47.48 (±19.81) R4u+ + 141.78 (± 36.53) R7u+ + 44.17 (± 17.80) R3v+ − 1.73 (± 1.37) (1)

(n = 20; r^2^ = 0.87; s = 0.32; F = 26.04; Q^2^_cv_ = 0.78; SPRESS = 0.36) (2)

The descriptors of the DRAGON [27,28] in the best model were:

- H7m–related to the probability of interaction between atoms and topological distance 7.
- R4u+, R7u+ and R3v+–related to the maximum steric contributions to the molecule shape related to the atoms with topological distance of 4, 7 and 3, respectively. These descriptor have greater values for higher influence on the shape and the lower geometric distance between them.

The evaluation set was made by five compounds, rationally selected as described by Golbraikh and colleagues [29]. Table 6 presents the calculated pIC_50_ values for the set of assessment, considering the descriptors of the selected QSAR model for the training set, the experimental pIC_50_ values, the residues values (difference between the values of calculated and observed activity) and the standard deviation (SD) of the mean of the residues. The compound that showed residues value greater than the average deviation was not well predicted by the selected model.

**Table 6:** pIC_50_ values, residues and standard deviation (SD) of the mean of the residues of the tested series.

pIC_50_ calculated	pIC_50_ observed	Residues	SD
2.54	2.38	0.16	0.26
3.87	3.70	0.17	
3.72	3.67	0.05	
2.28	1.53	0.75	
2.27	2.09	0.18	

**Figure 7:**
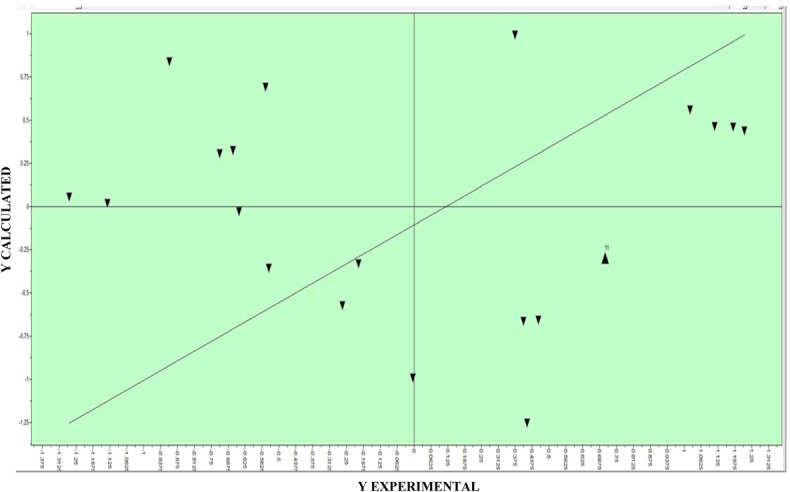
Best fit model obtained in PLS.

The partial least squares regression (PLS), was applied to the same set with twenty compounds. The PLS analysis, with five LVs ad 580 descriptors selected, yields a model with an r^2 ^of 0.91 and a q^2^_cv_ of 0.77 after variable selection via fractional factorial design (FFD) (Table 7 and Figure 7). The internal validation (cross-validation test) of PLS model was performed by the leave-one-out (LOO).

The Pentacle software is a computational tool for computing alignment-free molecular descriptors, also called GRid-INdependent descriptors or GRIND [30]. The software is based on Molecular Interaction Fields, describe the ability of the molecules to interact with other molecules and do not require to superimpose the compounds. After treatment of the descriptors with CLACC algorithm, we observed that the auto-correlogram of the probe shape TIP-TIP produced variables of greater values in active compounds, emphasizing physical and chemical characteristics, mainly steric, similar to the QSAR model.

**Table 7:** Statistical parameters of PLS model.

LV	SDEC	SDEP	R^2^	Q^2^
5	0.18	0.48	0.91	0.77

## 3. Experimental

### 3.1. Antifungal Activity

The *in vitro* antifungal activities of compounds **1**–**50** were investigated with regard to two species of pathogenic fungi, *Candida krusei* LM08 and *Criptococcus neoformans* ICB59. These strains were supplied by the URM Culture Collection of the Department of Mycology, Department of Pharmaceutical Sciences of the Federal University of Paraíba, Brazil. MIC values were determined by the microdilution broth method using microdilution plates according to the guidelines of the National Committee for Clinical and Laboratory Standards (NCCLS) [31]. Tests were performed according to Nascente *et al.* [32].

All strains were stocked in mineral oil at 18 °C. Viability tests and subsequent taxonomic confirmations were carried out according to Barnett *et al.* [33]. To obtain an inoculum of 2.5 × 10^3^ cells/mL, each strain was cultured in a tube containing 20 mL of Sabouraud Dextrose Agar plus yeast extract at 35 °C for two days. Subsequently, suspensions were prepared in sterile physiological solution (0.85%) and mixed in a rotator shaker. The inoculum was adjusted to 90% transmittance at 530 nm (measured via a spectrophotometer). Stock solutions of tested compounds were freshly prepared and dissolved in dimethylsulfoxide (DMSO), aliquoted and stored at −20 °C in stock solution with a final concentration of 1,024 μg/mL. Decimal dilutions of thiophene stock solutions were prepared in RPMI 1640 (Sigma Chemical Co., St., Louis, MO, USA) and buffered to pH 7.0 with 0.165 M morpholinopropanesulphonic acid (MOPS, Sigma). Microdilution plates containing serial dilutions of each compound (from 1,024 µg/mL to 1 µg/mL and from 10,000 µg/mL to 1,250 µg/mL (for the inactive compounds in the first serial dilution)) were inoculated with each organism; each plate included positive controls (fungi without a compound) and a negative control (medium only). The microdilution plates were incubated at 35 °C and were read visually after 24 and 72 h of incubation. For each strain, the MIC was defined as the lowest concentration of antifungal agent that completely inhibited the growth of the organism, as detected by the unaided eye and compared to the control group. All tests were performed in duplicate, and the results were expressed as the geometric mean of the MIC values obtained in the two trials [34,35].

### 3.2. Molecular Modelling

Using the program Hyperchem v. 8.0 [36], the chemical structures of the compounds of interest were drawn, and their geometry was optimised using MM+ force field [37]. Afterwards, we performed a new geometry optimisation based on the semi-empirical method AM1 (Austin Model 1) [38]. The optimised structures were subjected to conformational analyses using the random search method [20,21] with 1,000 interactions, 100 cycles of optimisation, and 10 conformers of lowest minimum energy. The selected dihedrals were evaluated by rotation in accordance with the standard (default) conditions of the program, in which the number of simultaneous variations was 1 to 8, acyclic chains were submitted to rotations from 60 to 180° and torsion rings were in the range of 30 to 120°.

The conformers of lowest minimum energy were selected, saved as .mol and exported to the Spartan 8 program [39]. The lowest energy conformers were then saved in .sdf format and imported into VolSurf+ for Windows [25,26]. They were subjected to the GRID force field, and interaction maps with the following “probes” were generated: H2O (light blue), O (red), N (dark blue) and DRY (green).

### 3.3. Chemometrics

The structures modelled as described above were used as the initial structures to calculate the molecular descriptors via the VolSurf+ program [25,26]. PCA and CPCA methodologies were applied to the set of interest using the VolSurf+ software [40,41,42,43,44,45].

### 3.4. QSAR

The conformers of lowest minimum energy obtained in the molecular modelling were exported to the DRAGON 5.4 program [27]. The selection of the descriptors was performed by multiple linear regression with the genetic algorithm GA-VSS of the MobyDigs 1.1 program [46]. Twenty compounds were used as training set and five compounds were used as a test (all of them rationally selected).

### 3.5. PLS

The conformers of lowest minimum energy obtained in the molecular modelling were imported by the Pentacle 1.5 program [30]. The partial least squares regression (PLS) was applied to a set of twenty compounds. The treatment of the descriptors was done with CLACC algorithm, using the Pentacle program.

## 4. Conclusions

Studies regarding the density functional theory emphasised the influence of water solubilities on the antifungal activities (with respect to strains of *Candida krusei* and *Criptococcus neoformans*) of 2-aminothiophene derivatives. CPCA and PCA analyses corroborated these results. The chemometric tools applied in this study generated good exploratory results. The VolSurf+ descriptors showed that hydrophilic properties are strongly correlated to the biological data. Our studies using Dragon and Pentacle programs emphasized the importance of the form and steric parameters for the antifungal activity. 

## References

[1] Brown J.M. (2004). Fungal infections in bone marrow transplant patients.. Curr. Opin. Infect. Dis..

[2] Bergold A.M., Georgiadis S. (2004). New antifungic drugs: A review. Visão Acadêmica.

[3] Ram V.J., Goel A., Shukla P.K., Kapil A. (1997). Synthesis of thiophenes and thieno[3,2-c]pyran-4-ones as antileishmanial and antifungal agents.. Bioorg. Med. Chem. Lett..

[4] Chobot V., Buchta V., Jahodárová H., Pour M., Opletal L., Jahodár L., Harant P. (2003). Antifungal activity of a thiophenepolyine from Leuzeacarthamoides.. Phytotherapy.

[5] Wardakhan W.W., Louca N.A., Kamel M.M. (2007). The reaction of 2 Aminocyclohexeno[b]thiophene derivatives with ethoxycarbonylisothiocyanate: Synthesis of fused thiophene derivatives with antibacterial and antifungal activities.. Acta Chim. Slov..

[6] Shaaban M.R., Saleh T.S., Farag A.M. (2009). Synthesis and antimicrobial evaluation of new thiophene and 1,3,4-thiadiazole derivatives..

[7] Islor A.M., Kalluraya B., Pai K.S. (2010). Synthesis, characterization and biological activities of some new benzo[b]thiophene derivatives.. Eur. J. Med. Chem..

[8] Fokialakis N., Cantrell C.L., Duke S.O., Skaltsounis A.L., Wedge D.E. (2006). Antifungal activity of thiophenes from Echinops ritro. J. Agric. Food Chem..

[9] Pinto E., Queiroz M.J.R.P., Vale-Silva L.A., Oliveira J.F., Begoin A., Begoin J.M., Kirsch G. (2008). Antifungal activity of synthetic di(hetero)arylamines based on the benzo[b]thiophene moiety. Bioorg. Med. Chem..

[10] Mendonça Junior F.J.B., Lima-Neto R.G., de Oliveira T.B., Lima M.C.A., Pitta I.R., Galdino S.L., da Cruz R.M.D., de Araújo R.S.A., Neves R.P. (2011). Synthesis and evaluation of the antifungal activity of 2-(substituted-amino)-4,5-dialkyl-thiophene-3-carbonitrile derivatives.. Lat. Am. J. Pharm..

[11] Abi-Said D., Anaissie E., Uzum O., Raad I., Pinzcowski H., Vartivarian S. (1997). The epidemiology of hematogenouscandidiasis caused by different Candida species.. Clin. Infect. Dis..

[12] Berrouane Y.F., Hollis R.J., Pfaller M.A. (1996). Strain variation among and antifungal susceptibilities of isolates of Candida krusei.. J. Clin. Microbiol..

[13] Pfaller M.A., Diekema D.J., Gibbs D.L., Newell V.A., Nagy E., Dobiasova S., Rinaldi M., Barton R., Veselov A. (2008). Global Antifungal Surveillance Group. Candida krusei, a multidrug-resistant opportunistic fungal pathogen: Geographic and temporal trends from the ARTEMIS DISK antifungal surveillance program, 2001 to 2005. J. Clin. Microbiol..

[14] Merz W.G., Karp J.E., Schron D., Saral R. (1986). Increased incidence of fungemia caused by candida krusei. J. Clin. Microbiol..

[15] Cheung M.C., Rachlis A.R., Shumak S.L. (2003). A cryptic cause of cryptococcal meningitis.. Can. Med. Assoc. J..

[16] Wormley F.L., Perfect J.R., Steele C., Cox G.M. (2007). Protection against Cryptococcosis by Using a Murine Gamma Interferon-Producing *Cryptococcus neoformans* Strain. Infect. Immun..

[17] Beebe K.R., Pell R.J., Seasholtz M.B. (1998). Chemometrics: A Practical Guide.

[18] Sharaf M.A., Illman D.L., Kowalski B.R. (1986). Chemometrics.

[19] Westerhuis J.A., Kourti T., Macgregor J.F. (1998). Analysis of multiblock and hierarchical PCA and PLS models.. J. Chemometr..

[20] Cohen N.C. (1996). Guidebook on Molecular Modeling in Drug Design.

[21] Leach A.R. (2001). Molecular Modeling: Principles and Applications.

[22] de Oliveira T.B. (2010). Síntese, elucidação estrutural e avaliação biológica de novos derivados tiazolidínicos e cicloalquil-tiofênicos. M.Sc. Thesis, Graduate Program in Pharmaceutical Sciences.

[23] Souza B.C.C. (2011). Síntese e Avaliação das atividades Antifúngicas e Antitumorais de novos derivados 2-[(arilideno)amino]-cicloalquil[b]tiofenos-3-carbonitrila. M.Sc. Thesis. Graduate Program in Pharmaceutical Sciences.

[24] Abu-Hashem A.A., El-Shehry M.F., Badria F.A. (2010). Design and synthesis of novel thiophenecarbohydrazide, thienopyrazole and thienopyrimidine derivatives as antioxidant and antitumor agents. Acta Pharm..

[25] Cruciani G., Crivori P., Carrupt P.-A., Testa B. (2000). Molecular fields in quantitative structure-permeation relationships: The VolSurf approach.. J. Mol. Struct..

[26] Cruciani G., Pastor M., Guba W. (2000). VolSurf: A new tool for the pharmacokinetic optimization of lead compounds.. Eur. J. Pharm. Sci..

[27] (2006). Dragon, Version 5.4;.

[28] Todeschini R., Consonni V. (2000). Handbook of Molecular Descriptors.

[29] Golbraikh A., Shen M., Xiao Z., Xiao Y.-D., Lee K.-H., Tropsha A. (2003). Rational selection of training and test sets for the development of validated QSAR models.. J. Comput. Aid. Mol. Des..

[30] Pastor M., Mclay I., Pickett S., Clementi S. (2000). Grid-Independent descriptors (GRIND): A novel class of alignment-independent three-dimensional molecular descriptors.. Med. Chem..

[31] (2002). National Committee for Clinical and Laboratory Standards; Method M27-A2;.

[32] Nascente P.S., Meinerz A.R.M., Faria R.O., Schuch L.F.D., Meireles M.C.A., de Mello J.R.B. (2009). CLSI broth microdilution method for testing susceptibility of *Malassezia pachydermatis* to thiabendazole. Braz. J. Microbiol..

[33] Barnett J.A., Paire R.W., Yarrow D. (2000). Yeasts: Characteristics and Identification.

[34] Eloff J.N. (1978). A sensitive and quick microplate method to determine the minimal inhibitory concentration of plant extracts for bacteria.. Planta Med..

[35] Souza E.L., Stamford T.L.M., Lima E.O., Trajano V.N. (2007). Effectiveness of *Origanum vulgare* L. essential oil to inhibit the growth of food spoiling yeasts. Food Control.

[36] (2009). HyperChem, Version 8.0.

[37] Allinger N.L. (1977). A hydrocarbon force-field utilizing V1 and V2 torsional terms.. J. Am. Chem. Soc..

[38] Dewar M.J.S.E., Zoebisch G., Healy E.F., Stewart J.J.P. (1985). AM1: A new general purpose quantum mechanical molecular model. J. Am. Chem. Soc..

[39] (2008). Spartan Model, Version 8.0;.

[40] Zamora I., Oprea T., Cruciani G., Pastor M., Ungell A.-L. (2003). Surface descriptors for protein-ligand affinity prediction.. J. Med. Chem..

[41] Crivori P., Cruciani G., Carrupt P.-A., Testa B. (2000). Predicting blood-brain barrier permeation from three-dimensional molecular structure.. J. Med. Chem..

[42] Kovatcheva A., Golbraikh A., Oloff S., Xiao Y.-D., Zheng W., Wolschann P., Buchbauer G., Tropsha A. (2004). Combinatorial QSAR of ambergris fragrance compounds.. J. Chem. Inf. Comp. Sci..

[43] Oprea T.I., Zamora I., Ungell A.-L. (2002). Pharmacokinetically based mapping device for chemical space navigation.. J. Comb. Chem..

[44] Cruciani G., Pastor M., Mannhold R. (2002). Suitability of molecular descriptors for database mining. A comparative analysis.. J. Med. Chem..

[45] Cianchetta G., Mannhold R., Cruciani G., Baroni M., Cecchetti V.J. (2004). Chemometric studies on the bactericidal activity of quinolones via an extended volsurf approach. J. Med. Chem..

[46] (2009). MobyDigs, Version 1.1;.

